# A Rare Presentation of Guillain-Barré Syndrome: A Case Report and Literature Review

**DOI:** 10.7759/cureus.95310

**Published:** 2025-10-24

**Authors:** Ivonne De la Hoz, Grayson White, Mohamad Sharbatji

**Affiliations:** 1 Internal Medicine, AdventHealth Orlando, Orlando, USA

**Keywords:** acute inflammatory demyelinating polyneuropathy (aidp), bickerstaff's brainstem encephalitis (bbe), facial diplegia, gait ataxia, guillain-barré syndrome, miller fisher syndrome (mfs), polyneuropathy

## Abstract

Guillain-Barré syndrome (GBS) is a term that includes a group of autoimmune-mediated peripheral neuropathies affecting varying nerve distributions, typically characterized by an acute onset of areflexic paralysis with albumino-cytologic dissociation. Given the wide range of subtypes and clinical presentations, GBS can be difficult to diagnose; however, the life-threatening nature of the disorder makes prompt diagnosis and treatment a top priority.

A 36-year-old athletic young male with a history of recent upper respiratory infection presented to the emergency department complaining of difficulty walking. He initially developed numbness in his genital and perianal area, followed by tingling in his hands and feet, and then difficulty walking due to unsteadiness, which ultimately prompted him to seek medical attention. His blood pressure was 142/85 mmHg, and his heart rate was 58 bpm. The initial physical exam revealed hyporeflexia and ataxic gait, while no deficits were observed in the cranial nerves, and the motor strength was intact in all four extremities. The following day, right-sided facial paralysis and proximal lower extremity weakness (able to move against only minimal resistance) were noted. Due to a strong suspicion of GBS, a five-day course of intravenous immunoglobulin (IVIG) was started. By the following day, the patient showed areflexia with worsening proximal lower extremity weakness and proximal upper extremity weakness. Cerebrospinal fluid (CSF) analysis showed albumin-cytologic dissociation. The total protein was elevated while the cell count was normal, findings consistent with GBS. By hospital day four, facial diplegia was evident, though notably, without any oculomotor involvement. In addition, he had developed dysarthria and dysphagia. The weakness in the lower extremities continued to progress, ultimately restricting movement only to instances when gravity was counterbalanced. Fortunately, the patient did not exhibit any signs of respiratory compromise, and both vital capacity (VC) and negative inspiratory force (NIF), which were carefully monitored, remained within normal limits. Upon completion of the IVIG course, the patient did not show any new neurologic deficits; instead started to show slow improvement in motor function. He continued to engage in intensive physical and occupational therapy and was eventually discharged to an inpatient rehab facility, where gradual improvement continued.

This patient exhibited a unique constellation of symptoms, combining features of classic GBS and rarer variants, posing a true diagnostic challenge. This case was marked by ataxia, facial diplegia, and paresthesia, in addition to the hallmark ascending weakness, highlighting the clinical complexity. GBS is a life-threatening condition; therefore, early recognition is crucial. Patients need close cardiac and respiratory monitoring as they are at risk for autonomic dysfunction and respiratory compromise. Treatment options include plasmapheresis and IVIG, and both therapies are equivalent and should be started rapidly.

## Introduction

Guillain-Barré syndrome (GBS) is a well-known cause of acute-onset flaccid paralysis. Despite broad general knowledge of the disorder, cases are rare with a worldwide incidence that varies from 0.4 to 2 per 100,000 people depending on geographic regions [1]. Classically, the disease is characterized by ascending symmetrical paralysis with hyporeflexia or areflexia. However, GBS has many subtypes with varying clinical presentations, which can further complicate diagnosis. The two most common subtypes include acute inflammatory demyelinating polyneuropathy (AIDP), which features classic sensorimotor symptoms with cranial nerve involvement and autonomic dysfunction, and acute motor axonal neuropathy (AMAN), which is a purely motor type of GBS. Even less frequently seen is the subtype known as Miller Fisher syndrome (MFS), presenting with a triad of ataxia, ophthalmoplegia, and areflexia [2,3].

Regardless of the clinical presentation, two-thirds of patients will often report a history of recent respiratory or gastrointestinal illness preceding symptom onset [4]. Diagnosis is made with history, clinical symptoms, and laboratory workup. Prompt lumbar puncture should be performed when GBS is suspected. Analysis of cerebrospinal fluid will show albumin cytologic dissociation (elevated protein levels with normal cell count), though it is not required for diagnosis. Additionally, some subtypes of GBS have specific antibodies that can be tested for, such as GQ1b and GT1a in MFS [5].

Prompt diagnosis is of the utmost importance in GBS, as without treatment, there is the risk of autonomic dysfunction and respiratory failure. Therefore, patients require careful cardiac monitoring and serial measurement of vital capacity (VC) and negative inspiratory force (NIF) to monitor the need for intubation and mechanical ventilation [6]. Given potential life-threatening complications, treatment should be initiated urgently. Plasmapheresis and intravenous immunoglobulin (IVIG) are both therapeutic options, though one treatment is not preferred over the other [7].

## Case presentation

A 36-year-old athletic young male presented to the emergency department complaining of difficulty walking. He had a recent history of an upper respiratory infection two weeks before, which had resolved spontaneously.

The patient had developed numbness in his genital and perianal area, but he denied any fecal or urinary incontinence. In addition, he developed tingling in his hands and feet and difficulty walking due to unsteadiness, which ultimately prompted him to seek medical attention. His blood pressure was 142/85 mmHg, and his heart rate was 58 bpm(Table 1). The initial physical exam revealed decreased deep tendon reflexes and ataxic gait, while no deficits were observed in cranial nerve examination, and the motor strength was intact in all four extremities. The complete blood count and comprehensive metabolic panel were unremarkable.

**Table 1:** Initial vital signs at presentation mmHg: millimeters of mercury; bpm: beats per minute; rpm: respirations per minute; F: Fahrenheit.

Admission vital signs		
Parameter	Reference range	Value
Blood pressure	<120/80 mmHg	142/85 mmHg
Heart rate	60-100 bpm	58 bpm
Respiratory rate	12-20 rpm	16 rpm
Oxygen saturation	95-100%	100%
Temperature	97.5-99.5 F	97.5 F

The following day, right-sided facial paralysis and proximal lower extremity weakness (able to move against minimal resistance only) were noted. Of note, no oculomotor involvement was appreciated. Due to a strong suspicion of GBS, a five-day course of IVIG was started at a dose of 0.4 grams/kg daily. By the following day, the patient showed areflexia with worsening proximal lower extremity weakness and new onset proximal upper extremity weakness (able to move against gravity and moderate resistance).

**Figure 1:**
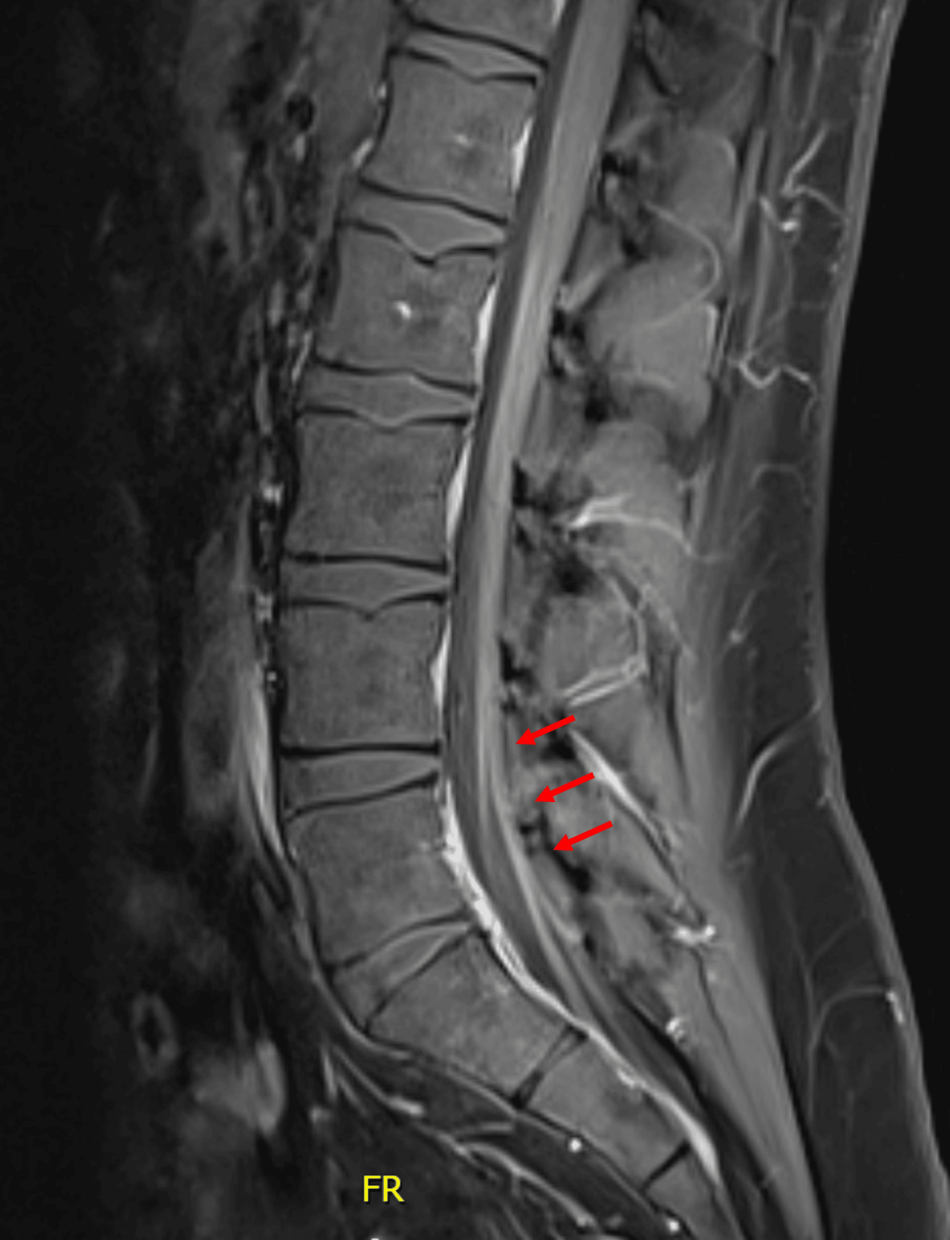
MRI with contrast of the lumbar spine (sagittal view). Note abnormal smooth enhancement along the cauda equina (arrows).

A lumbar puncture was performed, and the cerebrospinal fluid (CSF) analysis showed albumin cytologic dissociation. The total protein was 313 mg/dL (reference range = 15-45 mg/dL), and the white cell count was 2 u/L (reference range = 0-5 u/L), findings consistent with GBS (Table 2). Magnetic resonance imaging (MRI) of the lumbar spine with intravenous contrast showed abnormal smooth enhancement along the cauda equina, a finding that could be seen in GBS and other infectious or inflammatory polyradiculopathy syndromes (Figure 1).

**Table 2:** CSF analysis​. Results of CSF analysis. Note significant elevation in protein and normal nucleated cells, consistent with albumino-cytologic dissociation. CSF: cerebrospinal fluid; mg/dl: milligram/deciliter; uL: microliter; IV: ImmunoValue.

Cerebrospinal fluid analysis		
Parameter	Reference range	Result
Color	Colorless	Colorless
Total protein	15-45 mg/dL	313 mg/dL
Nucleated cells	0-5/uL	2/uL
White blood cells	0-5 /uL	2/uL
Red blood cells	<0/uL	29/uL
GQ1b antibody, IgG	0-50 IV	3 IV

**Figure 2:**
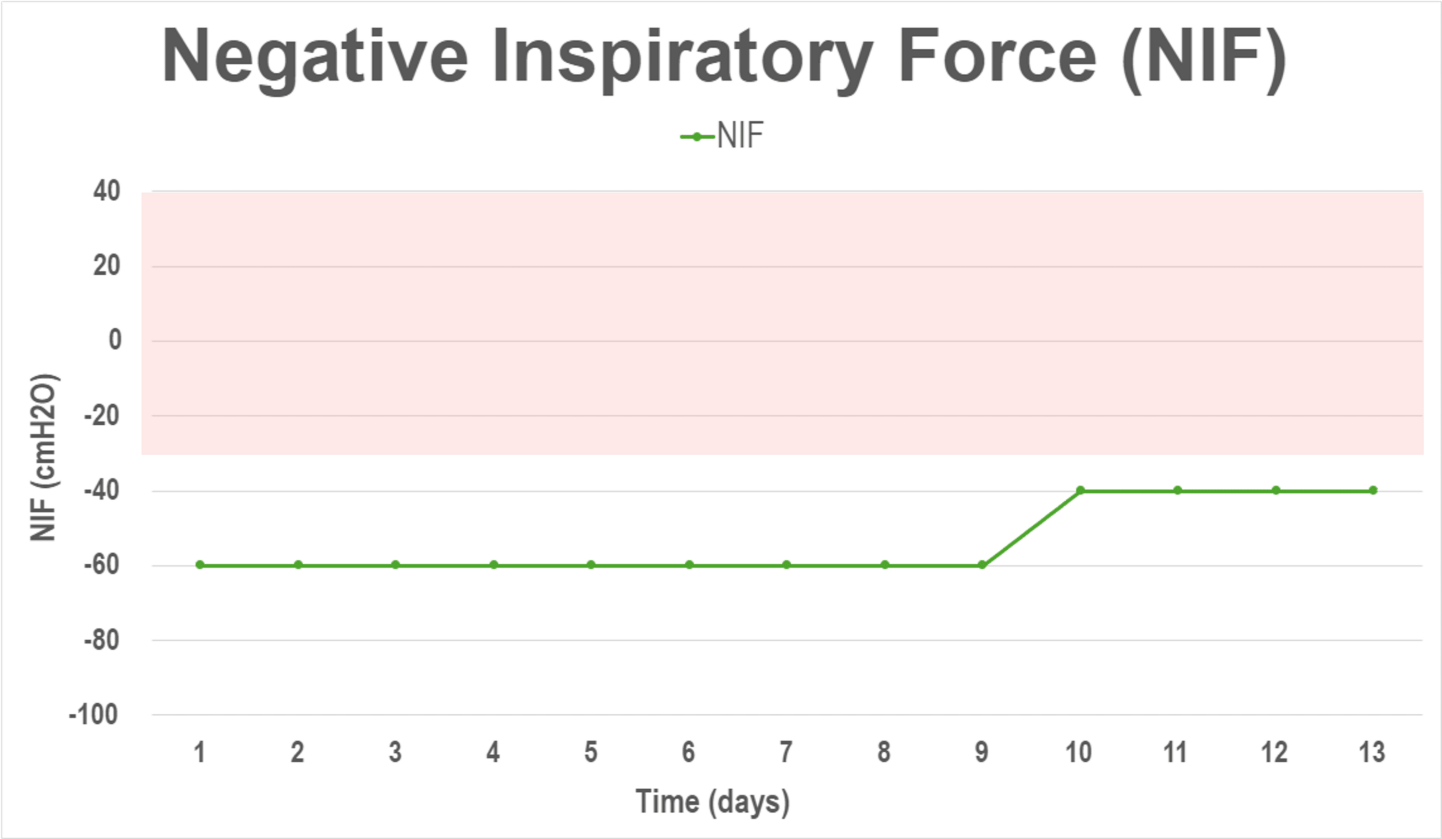
Negative inspiratory force (NIF) trend during hospital stay. Note the trend of the negative inspiratory force (green). A NIF less than -60 cmH2O is considered normal, while a value equal to or more than -30 cmH2O suggests respiratory muscle weakness and impending respiratory failure (shaded red). cmH2O: centimeters of water.

By hospital day four, facial diplegia was notorious. In addition, he developed dysarthria, difficulty chewing food, and dysphagia. Simultaneously, weakness in the lower extremities continued to progressively worsen (able to move only when gravity was counterbalanced). Fortunately, the patient did not exhibit any signs of respiratory compromise, and both vital capacity (VC) and negative inspiratory force (NIF), which were carefully monitored, remained within normal limits (Figure 2).

After completing the course of IVIG, the patient did not show any new neurologic deficits, and he started to show mild improvement in motor function. He continued to work intensively with physical and occupational therapy and was discharged to an inpatient rehab facility, where he continued to show signs of improvement and ultimately went home after a one-month stay. After six months, he was able to ambulate independently.

## Discussion

This patient exhibited a unique constellation of symptoms with overlapping features of the classic presentation and rare variants of Guillain-Barré, including ataxia, facial diplegia, and paresthesia, and later developed the hallmark ascending weakness, highlighting the clinical complexity and ultimately posing a true diagnostic challenge.

Bilateral facial palsy or facial diplegia is a rare neurologic finding that has been reported as a prominent sign of a GBS variant, the facial diplegia with paresthesia variant, described as a localized form of AIDP [8]. Susuki et al. conducted a retrospective review of patients diagnosed with a GBS variant presenting with facial diplegia and limb paresthesias, and only 18% of the cases had concomitant limb weakness, which was mild in all instances, contrary to our case, where the patient developed disabling paraparesis. It is worth noting that facial diplegia is often associated with sarcoidosis and Lyme disease, and both entities should be considered as differential diagnoses [9].

In the case presented, the patient exhibited ataxia early on; this neurologic sign has been associated with Guillain-Barré variants, traditionally with the MFS, where patients present additionally with intact strength, areflexia, and ophthalmoplegia [2,8]. Similarly, Bickerstaff’s brainstem encephalitis (BBE), a central variant of MFS syndrome, also presents ataxia and ophthalmoplegia, but with associated altered mental status, which is the hallmark. Both entities, classic MFS and BBE variants, are associated with IgG antibodies to GQ1b, but negativity does not exclude the disease [8,10].

Wakerley et al. retrospectively analyzed 103 patients who met diagnosis criteria for GBS in a span of 15 years, concluding that classic GBS and classic MFS were seen in 71% and 17% of the cases, respectively, while patients with overlapping syndromes represented less than 3% of the sample [3].

The diagnosis of GBS is clinical. However, certain findings can support the diagnosis and become particularly helpful when the presentation is atypical. CSF analysis reveals albumin cytologic dissociation in up to 75% of GBS cases; however, this finding can be absent in nearly half of the cases if lumbar puncture is performed within the first week [2,8].

Nerve conduction studies allow confirmation of neuropathy. However, they are less practical, often unavailable, and therefore rarely performed. On the other hand, imaging studies are often obtained, primarily to exclude conditions such as transverse myelitis or cauda equina syndrome, which can present similarly. Oftentimes, contrast MRI can demonstrate enhancement of nerve roots in the conus medullaris and cauda equina, which can be a sign of inflammatory neuropathy such as GBS. This radiologic finding was present in our patient; however, it is not specific [1,11].

The disease will usually plateau after 28 days, but the journey toward recovery is long and challenging. Most improvement will occur during the first year, but some patients may continue to recover for up to three years. Our patient was able to ambulate independently after six months; unfortunately, this is not the case in 20% of the patients [4,8].

Treatment modalities include IVIG and plasma exchange, both proven effective and equivalent. The choice of treatment will typically depend on institutional policies and available resources [7]. Verboon et al. conducted a large multinational and multicenter retrospective review showing that patients with MFS and other GBS variants more commonly received IVIG, but it is worthwhile noting the lack of trials comparing the efficacy of IVIG and plasmapheresis in these rare variants [12].

## Conclusions

To conclude, this case underscores the critical importance of recognizing and understanding rare variants of GBS, as patients can present with atypical and overlapping features, such as those seen in this patient, challenging clinicians and increasing the risk of misdiagnoses and delayed treatment, which could lead to rapid clinical deterioration and life-threatening complications.
